# The Characterisation of Three Types of Genes that Overlie Copy Number Variable Regions

**DOI:** 10.1371/journal.pone.0014814

**Published:** 2011-05-26

**Authors:** Cara Woodwark, Alex Bateman

**Affiliations:** Wellcome Trust Sanger Institute, Wellcome Trust Genome Campus, Hinxton, Cambridge, United Kingdom; University of Minnesota, United States of America

## Abstract

**Background:**

Due to the increased accuracy of Copy Number Variable region (CNV) break point mapping, it is now possible to say with a reasonable degree of confidence whether a gene (i) falls entirely within a CNV; (ii) overlaps the CNV or (iii) actually contains the CNV. We classify these as type I, II and III CNV genes respectively.

**Principal Findings:**

Here we show that although type I genes vary in copy number along with the CNV, most of these type I genes have the same expression levels as wild type copy numbers of the gene. These genes must, therefore, be under homeostatic dosage compensation control. Looking into possible mechanisms for the regulation of gene expression we found that type I genes have a significant paucity of genes regulated by miRNAs and are not significantly enriched for monoallelically expressed genes. Type III genes, on the other hand, have a significant excess of genes regulated by miRNAs and are enriched for genes that are monoallelically expressed.

**Significance:**

Many diseases and genomic disorders are associated with CNVs so a better understanding of the different ways genes are associated with normal CNVs will help focus on candidate genes in genome wide association studies.

## Introduction

Detectable copy number variable (CNV) regions vary greatly in size, from macroscopic mutations, visible as gain or loss of heterochromatic bands, down to regions of a just a few kilobases. Recent developments in CNV detection have brought the field into even closer focus with CNVs as small as 500 nucleotides being detected [1]. These CNVs may be so small that they are entirely contained within a gene. Larger CNVs may cover whole genes and can be up to several megabases long. CNVs have been associated with many genomic disorders such as Charcot–Marie–Tooth disease, as well as diseases such as hypertension and schizophrenia and may also be associated with susceptibilities to certain cancers. [2].

It has been presumed that the expression of CNV genes would correlate with copy number, so that for example, a gene that has one allele duplicated (copy number of 3) would have 1.5 times the level of expression of the wild type copy number of 2. In other words an expression ratio of 1.5 (3∶2).

Stranger *et al.* [3] showed that SNPs are responsible for 83.6% of the detectable variation in expression among individuals compared with only 17.7% that is due to CNVs despite the fact that CNVs cover a greater proportion of an individuals genome than is covered by SNPs. Some of this variation in expression is due to CNVs that lie upstream of a gene disrupting the regulatory regions. Stranger *et al.* [3] agreed with recent work in this lab by Schuster-Böckler *et al.* [4] that there is a significant difference in the expression ratios between duplicated and deleted genes, and that this difference is much smaller than expected. A similar result has also been found in mouse [5]. However, there were a number of caveats that may have led to these results, including the fact that the precise CNV breakpoints were unknown so that it was possible that genes were being included that were not truly within the CNV, and *vice versa,* which would bias the mean expression levels. Not knowing the precise breakpoints might also lead to the inclusion of genes which partially overlap the CNV. In this case only a part of the gene is in multiple copy, so that a supposed copy number of 3 may be only one functional allele and two disrupted alleles. This would also bias average expression ratios for amplified genes. However, recent work by Conrad *et al*. [1] has produced a new dataset of CNVs with breakpoints that are claimed to be accurate to within 60 nucleotides. This improvement in the accuracy of break point prediction allows us to say with a much greater degree of certainty that a gene is fully between the breakpoints of a CNV or not.

This work uses the Conrad *et al*. [1] CNV coordinates for those individuals that we have expression data for from Stranger *et al.* [4]. Only Ensembl protein coding genes where the coding region or UTR of the gene overlaps with a CNV are included. The accuracy of the predicted CNV breakpoint coordinates from Conrad *et al.* [1] means that we can be reasonably confident that only the genes that fall entirely within CNVs, will be included when testing the correlation of expression with copy number. The characteristics and potential mechanisms for the regulation of expression of these genes is investigated and compared to two other categories of CNV associated genes (described below).

## Results

### Classification of CNV Associated Genes

**Figure 1:**
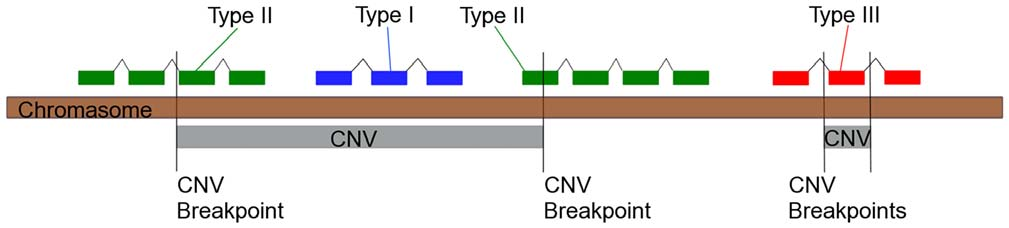
Schematic illustrating the three different categories of CNV genes. Type I genes vary in copy number whilst type II and III genes have the wild type copy number. Type III genes contain the CNV region within the gene. The CNV may overlap an intron, exon, or UTR. Here the CNV is shown overlapping an exon. Type II genes overlap with the CNV region. In the example shown here, the CNV break points lie within two different genes. In this example fusion genes would be created by the CNV. It is more common though for just the 5′ or 3′ end of the gene to be lost or duplicated.

The genes that vary in copy number and that are contained entirely within the CNV are the most obvious class of genes to consider when talking about CNV associated genes. We classified these genes as type I CNV genes (blue gene in figure 1). However, there are two other possible structural relationships between gene and CNV. The first of these is where one CNV break point is found within a gene so that the gene partially overlaps the CNV. We classified these genes as type II (green genes in figure 1). These type II genes are often disrupted and may even form fusion genes. For example, the two type II genes in figure 1 might form a fusion gene if the CNV were deleted or duplicated in situ. Fusion genes, although rare, are thought to occur most frequently between paralogues. Type III genes are those that contain the CNV within the gene. These genes may also be disrupted depending on whether the CNV excises exons that are in phase or not. Overall, type II and III genes will have the wild type copy number and are therefore expected to have the wild type expression unless the gene (or it's regulatory regions) are disrupted by the CNV.

### CNV Gene Characteristics

In order to better understand the biology of these three types of CNV genes we looked at a few basic characteristics, such as, mutation rates, possible selective pressures and the type of genes involved. There are 420 type I genes, 378 type II and 1172 type III genes (table 1). Type one genes are shorter on average (19,291 nucleotides (nt)) than type II (58,362 nt) which are it turn much shorter than type III genes (236,909 nt). Which is not surprising when we think that type III genes contain CNVs whilst type I genes are contained within CNVs. However, the length of overlap between the CNV and the gene shows the opposite trend with type I genes overlapping on average by 19,291 nt, type II by 7,990 nt and type III by 4,731 nt.

**Table 1:** Mean and median values for length of genes, and CNV gene overlaps for the three types of CNV genes.

	Number	Mean (median) length	Mean (median) overlap	Mean (median) Percent overlap wrt the gene
Type I	420	19291 (8284)	19291 (8284)	100 (100)
Type II	378	58362 (25990)	7990 (2399)	27.962 (18.092)
TypeIII	1172	236909 (145109)	4731 (2195)	5.275 (1.804)

Type I genes appear to be evolving rapidly as measured by the median dN plus dS values. (See table 2.) The mean value is a little lower than that for type II genes but in this case just a small number of very rapidly evolving genes (or which were perhaps mislabelled as orthologues) have skewed the mean values. Some type I genes even appear to be under positive selection (mean dN/dS greater than one). Most of the type I genes in CNVs appear to show increased variation and mutation rates compared with the genome as a whole. Many, such as the MHC (HLA) genes are under density dependent balancing selection [6] due to their important role in the immune response's “arms race” against constantly varying pathogens. On the whole type I genes tend to be involved in the immune response, as may be seen from table 3, with three of the top four categories of genes being involved in various aspects of both the adaptive and the innate immune systems.

**Table 2:** Mean and median values for dN/dS and dN+dS for the three types of CNV genes and non CNV genes.

Gene type	dN/dSmean (median)	Kolmogorov-Smirnov Test Probability (direction of significance)	dN + dS mean (median)	Kolmogorov-Smirnov Test Probability (direction of significance)
**I**	1.1598 (0.4364)	1.113×10 ^−10^ ↑ ***	0.03450 (0.0250)	2.168×10 ^−11^ ↑ ***
**II**	0.6118 (0.2941)	0.01801 ↑ *	0.04161 (0.0226)	1.628×10 ^−8^ ↑ ***
**III**	0.3132 (0.2027)	0.001491 ↓ ***	0.02958 (0.0169)	0.04667 ↓ *
**Non CNV**	0.4522 (0.2308)	NA	0.03435 (0.0172)	NA

Probability values for the Kolmogorov-Smirnov test are given for the comparison of the CNV gene type and non CNV genes.

Significance is indicated by

*** =  p<0.005

** =  p<0.01

* =  p<0.05

Arrows show whether the CNV genes are evolving more rapidly than non CNV genes ↑ or more slowly ↓.

**Table 3:** DAVID Functional Annotation Clustering for CNV Genes.

CNV gene type	DAVID Functional Clusters	Enrichment Score
I	Immunoglobulin	31.7
I	MHC	19.84
I	Olfactory Receptor	4.62
I	Glutathionine S Transferase (GST)	2.71
I	hormone	2.53
I	Keratin	2.13
I	Chromatin Associated	1.31
II	APOBEC (mRNA editing)	1.66
II	Cell adhesion	1.53
II	Plexin fold (extracellular)	1.5
II	Keratin	1.47
III	plasma membrane	4.44
III	cell-cell signaling	3.9
III	synapse	3.75
III	ion binding	3.75
III	SH3 domain (extracellular)	3.66
III	Fibronectin (extracellular)	3.39
III	EGF (extracellular)	3.24
III	Nucleotide binding	3.09
III	Sushi (extracellular)	3.09
III	Cell adhesion	3.01

The type III genes tend to be involved in cell cell signalling and are enriched for extracellular domains such as SH3 (table 3). These genes appear to be mutating more slowly than genes in the rest of the genome as measured by either the mean or the median dN plus dS. The very low dN/dS values would appear to indicate that strong negative selection is acting on type III genes (table 2).

The properties of type II genes appear to lie somewhere between type I and III and share some gene functions type I (keratins) and type III (cell adhesion) genes (table 3). The mutation rate and selective pressures also occupy the middle ground and as such give the appearance of not being significantly different from non-CNV genes (table 2).

### Gene Expression

**Figure 2:**
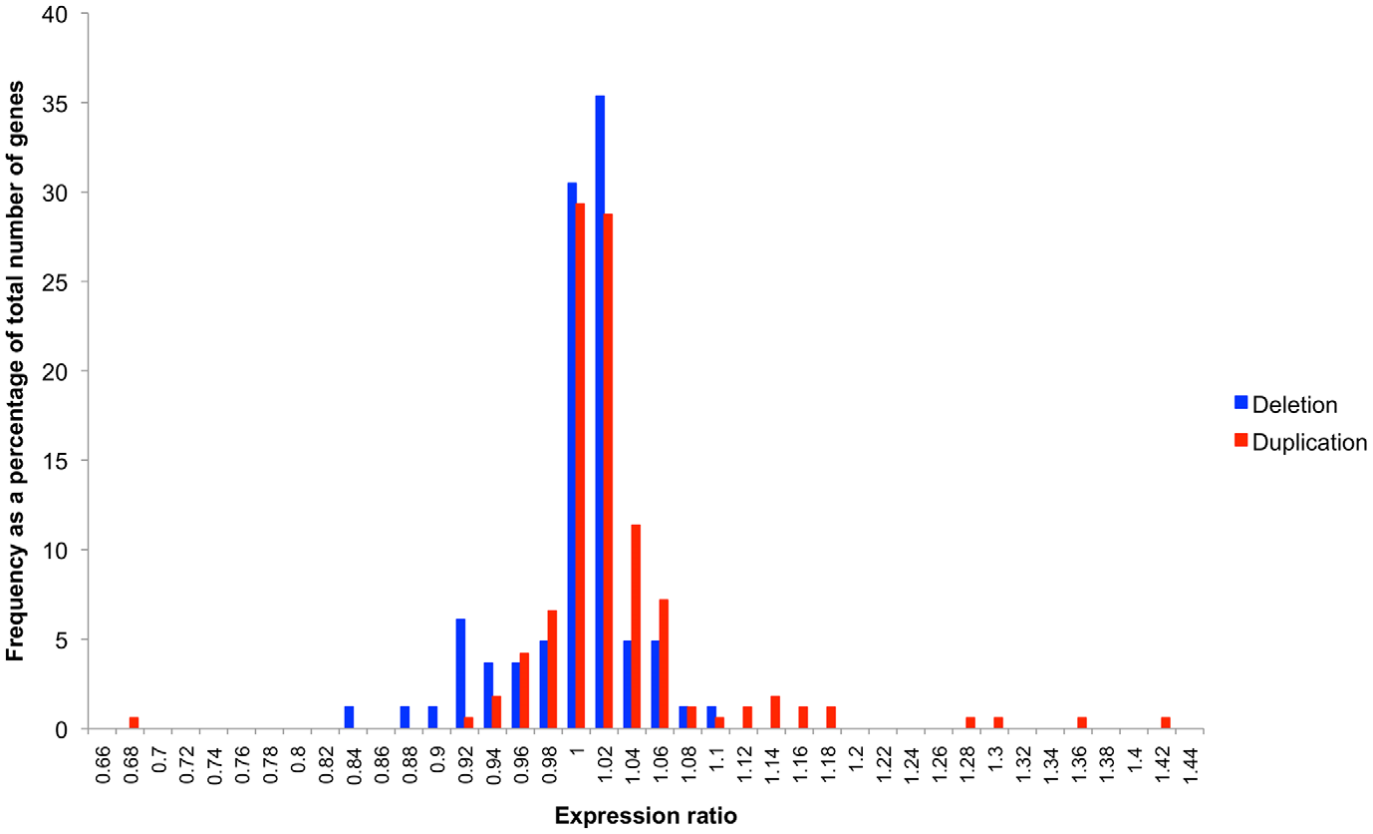
Frequency distribution graphs of expression ratios. Expression ratio distribution for the same individuals and the same expression data as analysed by Schuster-Böckler *et al.* [4]. Only type I genes based on the CNV coordinates from Conrad et. al. [1] were used.

**Figure 3:**
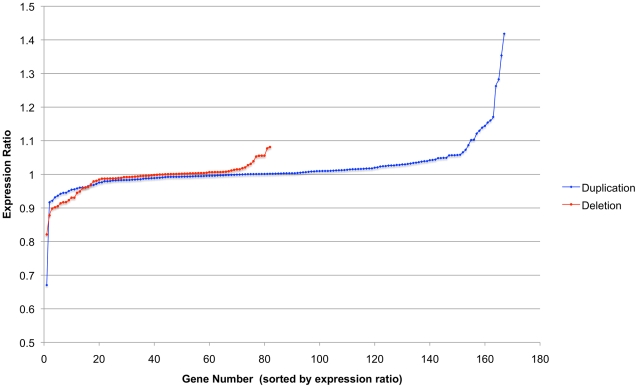
Expression ratio for each type I gene. Expression ratios (normalised expression) for each duplicated and deleted type I genes. Note that although there are more duplicated genes with higher expression ratios the vast majority of both duplicated and deleted genes have an expression ratio very close to one rather than the 0.5 (1 copy) or >1.5 (>3 copies) expected. These distributions are similar to those of Supplemental figure S1.

The expression ratio analysis of Schuster-Böckler *et al.* [4] was repeated with the Conrad *et al.* [1] CNV data set using only the type I genes. As figure 2 shows, despite the increased accuracy of these CNVs most genes, whether duplicated or deleted, have expression levels very similar to the wild type expression level (expression ratio of one) with a difference in means of just 0.026 between them. This may be seen more clearly in figure 3, which shows the expression ratios plotted for each type I gene. The graphs for both the duplicated and deleted genes show that most genes have an expression ratio of one (the straight horizontal stretch of the graph) with only a few genes at either end of the graph that have expression ratios that differ greatly from one. Similar shaped graphs are also produced for type II and III genes (Supplemental figure S1) which would imply that the copy number of the gene does not affect the expression of type I genes as only parts of the type II and III genes vary in copy number not the whole gene. This apparent lack of correlation of gene dosage on expression levels suggests that the type I genes must be under tight homeostatic regulation that somehow compensates for gene dosage. Although, the whole of type II and III CNV genes do not vary in copy number, their expression ratios do vary in a similar way to type I genes so may also require dosage compensation mechanisms to cover the affects of disrupted alleles or missing regulatory regions.

#### Dosage Compensation

Gene expression regulation and therefore, dosage compensation, is a complex process involving many different stages before, during and after transcription. Pretranscriptionally, the position of the chromosome within the nucleus [7], the chromatin conformation, [8] histone and DNA modifications, whether particular transcription factors, inhibitors or activators are bound can all affect whether a gene is transcribed [9]. During transcription even the speed of RNA polymerase II affects splicing which can alter the transcript produced and may even lead to a nonsense transcript being produced [10]. In this case post transcriptional regulation in the form of nonsense mediated decay would destroy the transcript so no gene product is produced [11]. Unfortunately, we still do not have the data to be able to check which, if any, of these particular methods are involved in dosage compensation. We do have the data to look at miRNA regulation, imprinting and monoallelic expression as mechanisms which may be involved in homeostatic regulation of gene product levels.

#### Monoallelic Expression

Dosage compensation by imprinted or monoallelically expressed genes has been well documented although we do not fully understand the mechanisms involved. Imprinted genes are thought to have one parental allele switched off by antisense non-coding RNAs [12]. Monoallelic genes may have either allele expressed irrespective of the parent of origin although the mechanism that controls which allele is expressed is not understood. If CNV genes are imprinted or monoallelically expressed this may explain why there is so little correlation of expression levels with copy number. The 160 imprinted genes [13] and the 525 experimentally verified monoallelically expressed genes from Gimelbrant *et al.* [14] were compared to the CNV genes. Imprinted genes tend to be expressed in a parent of origin fashion in specific tissues, such as the brain, or during development. Only seven imprinted genes are found in the experimentally verified monoallelically expressed data set. This is presumably due to fact that only the lymphoblastoid cell lines were tested.

Table 4 shows that imprinted genes are neither enriched nor underrepresented in all three gene types. However, type I genes show a paucity of monoallelically expressed genes whilst type III genes are significantly enriched for them. This result is interesting as it completely contradicts our hypothesis that monoallelic expression mechanisms might be a possible dosage compensation mechanism for type I genes.

**Table 4:** Observed and expected numbers of imprinted and monoallelically expressed genes for the three types of CNV gene.

	Imprinted genes (expected)	Monoallelic genes (expected)
Type I	2 (3.368)	1 (11.053) ***
Type II	3 (3.032)	9 (9.947)
Type III	15 (9.399)	129 (30.842)***

Significance levels indicated by ***  =  p<0.005.

#### miRNA Regulation

It is thought that regulation of expression by miRNAs represents a layer of homeostatic fine tuning that mops up excess transcripts by pairing with a target site in the 3′UTR of the gene and either destabilising the mRNA or marking it for decay (see Bartel [15] for review). There is also evidence that miRNAs may be able to activate transcription [16]. Li *et al*. [17] suggest that duplicated genes (paralogues) may be preferentially regulated by miRNAs, although, some current estimates predict that actually most human genes may have their expression regulated by miRNAs [18].

Regulation of expression by miRNAs seems to be a likely mechanism to have evolved to regulate type I genes as miRNAs recognise conserved “seed” sites in the 3′UTR which are unlikely to be disrupted in type I genes. Therefore, regulation of expression by miRNAs may be part of the homeostatic mechanism that underlies the cell's ability to dosage compensate for variable numbers of copies of a gene. Type II and III genes may be less likely to be regulated by miRNAs as the 3′UTRs may be disrupted in these gene classes.

To test the hypothesis that CNV type I genes have their expression regulated by miRNAs, an experimentally verified set of miRNA target genes [19] were mapped to the Ensembl gene set. Of the 384 genes mapped, only 5 were found to be type I genes, which is not significantly different from the 8 expected by chance (χ^2^ = 0.849, p = 0.357). It was felt that this dataset, although accurate as the genes were experimentally verified as being regulated by miRNAs, was too small as it is thought that most human genes may be regulated by miRNAs [18]. Two sets of predicted miRNA targets were obtained from miRGen [20] who have combined miRanda [21], PicTar [22] and TargetScanS [18] prediction methods (See Methods for details).

The first dataset analysed was a conservative “Intersection” dataset, where all three prediction methods must predict the same miRNA target for that gene miRNA pairing to be included. The second “Union” dataset counted all targets predicted by any of the three methods. Table 5 shows the results for these analyses. The Intersect set predicted that 15 of the type I genes would have their expression regulated by miRNAs. A χ^2^ test showed that this is significantly fewer than the 39.5 genes expected by chance (χ^2^ = 14.47, p = 0.00014). This paucity of miRNA regulated genes in the type I CNV genes, was also found for the Union dataset where 352 rather than 392.1 miRNA target genes were found (χ^2^ = 54.93, p = 1.25×10^−13^). (A more liberal Intersection, dataset that required any two of the three methods to predict a target for the gene for that gene to be included, also showed a significant lack of miRNA targeted genes in the type I genes. (57 genes rather than the predicted 158.2 expected; χ^2^ = 105.25 p<2.2×10^−16^).

**Table 5:** Summary of results for the three types of CNV genes for miRNA target analysis.

Gene type	Number of Genes	No of Genes with miRNA targets predicted for Intersect Data Set (Expected)	Mean no. of Intersect miRNAs predicted per gene (median)	No of Genes with MiRNA targets predicted by Union (Expected)	Mean no. of Union miRNAs predicted per gene (median)	Mean no. of Union targets per gene (median)	3′UTR Length (median)
I	420	***15 (39.500)	4.467 (3.000)	***352 (392.100)	***18.370 (12.500)	***24.320 (13.500)	***914 (561)
II	378	30 (33.745)	2.80 (2.000)	360 (352.875)	25.350 (20.000)	35.540 (24.000)	1299.000 (923.500)
III	1172	***151 (104.628)	4.179 (3.000)	***1133 (1094.102)	***27.940 (25.000)	***41.23 (31.00)	***1777 (1335)
Non CNV	17980	1585 (1605)	3.503 (2.000)	16779 (16784)	24.84 (20.000)	33.960 (25.00)	1230 (815)

Significance levels indicated by *** probability <0.005.

This lack of type I CNV genes potentially regulated by miRNAs was surprising but perhaps not so strange as the observation that type III genes appear to have a significant enrichment for genes predicted to be regulated by miRNAs. Type II genes again fall between type I and III and have nearly the expected number of genes regulated by miRNAs as predicted by both the Intersection and Union datasets. Results for both datasets and the three gene types are shown in table 5.

Even those type I genes that do have predicted miRNA targets are regulated by significantly fewer miRNAs than genes in the rest of the genome for the Union dataset (Kolmogorov-Smirnov test D  = 0.2347, p = 5.058×10^−11^). The type I genes were found to have significantly shorter 3′UTRs than genes in the rest of the genome with a mean of 914 nucleotides compared with 1266 for the rest of the genome (Kolmogorov-Smirnov test D  = 0.195, p = 1.965×10^−07^). As we know, a miRNA may have a number of targets in the same UTR so that a gene might be regulated by just one miRNA but have, for example, five targets sites for it in the 3′UTR while another gene may also have five targets but be regulated by three different miRNAs. While there is a correlation with number of targets and 3′UTR length there is no correlation between number of miRNAs regulating the gene and the 3′UTR length. There is a very significant positive correlation between the numbers of targets found in the 3′UTR (Pearson p-value <2.2×10^−16^) and the length of the 3′UTR but not with the number of miRNAs involved (p-value  = 0.2355). Therefore, although the type I genes do tend to have shorter 3′UTRs this is unlikely to be the reason for the paucity in miRNAs regulating the expression of these genes as there is no correlation with the number of miRNAs and the 3′UTR length.

Type III genes were found to have opposite characteristics to the type I genes. They have an enrichment for genes with predicted miRNA targets for both the Intersection and Union data sets, as seen in table 5. They also have significantly more predicted miRNAs per gene, as well as more target sites per gene and significantly longer 3′UTRs than the non-CNV genes. Type II genes have approximately the expected numbers of miRNA targets in both the Intersection and Union datasets. They also have similar numbers of predicted miRNAs and predicted targets as the non-CNV genes. Even the 3′UTR lengths display a similar distribution as non-CNV genes.

It should be noted that further subdividing the gene types by allele type depending on whether the CNV was normally a deletion, an amplification or could occur as both, had no affect. All three allele types appear to give similar results (results not shown).

## Discussion

The three categories of genes based on their physical association to a CNV appear to also have coherent biological themes. The results for the three categories were very different and often surprising with some being the opposite of what we had initially hypothesised. Here we will discuss the findings for each gene type in turn.

Ohno [23] suggested that although gene duplication does occur it is often deleterious due to unbalanced gene dosage. It is presumed that genes that vary in copy number would have expression levels that would depend on the copy number of the gene. The CNVs studied here belong to HapMap individuals and most are not thought to be associated with major diseases but are thought to be neutral mutations. However, it was still a surprise to discover that the expression of the genes associated with these CNVs was similar to that of the wild type copy number irrespective of copy number and whether the gene was entirely within the CNV as in type I or merely overlapped the CNV as in type II and III. This suggests that some sort of dosage compensation mechanism must be involved in the regulation of these genes.

MiRNAs might be a likely dosage compensation mechanism for type I genes as they are duplicated (or deleted) in their entirety thus maintaining intact 3′UTRs. However, this was not the case as type I genes showed a significant paucity of genes regulated by miRNAs and those genes that did have predicted miRNA targets had significantly fewer targets than expected even allowing for the short 3′UTRs of these genes. The shorter 3′UTRs and lack of regulation by miRNAs of these type I genes is similar to what has been found for housekeeping genes [24]–[26]. It is thought that house keeping genes have evolved short UTRs to avoid “accidental” regulation by miRNAs as it is easier for a single point mutation to create a new miRNA target (or lose one) than to create a new transcription factor biding site [27]. However, type I genes showed no significant enrichment for housekeeping genes (data not shown). For example, the MHCII genes which are enriched in type I genes, have evolved rapid mutation rates to keep up with the “arms race” in their fight against pathogens. MHCII genes lack miRNA targets and are all regulated by the Class II TransActivator (CIITA) which has a low affinity binding site that is difficult to disrupt by point mutation [28]. This hints that rapidly evolving type I genes are less likely to be regulated by miRNAs and have also evolved short 3′UTRs for the same reasons as house keeping genes despite the fact that housekeeping genes have low mutation rates.

The fact that genes such as the MHC genes that are under balancing selection in type I genes may be due to their requirement for ongoing variation as copy number variation is another method of increasing the potential for variation amongst individuals.

The type III genes are under very different evolutionary pressures than type I genes as they have evolved long UTRs and are significantly enriched for genes that are regulated by miRNAs. Their lower mutation rates mean that miRNA targets are less likely to be disrupted by point mutation. There is the possibility that the CNV itself might disrupt the UTR but most CNV breakpoints in type III genes are within the introns (nearly 90%) and are often associated with repetitive elements such as Alu [1], so the CNV is unlikely to disrupt the 3′UTR and it's miRNA targets (only nine of the 1172 type III genes had their 3′UTRs disrupted by the CNV). Also, CNVs that completely overlap the 3′UTR will be classified as type II genes.

The type III genes are significantly enriched for genes that are normally monoallelically expressed. Initially this result was rather surprising but recent work by Necsulea *et al*. [29] linking monoallelic expression with recombinant hotspots may help to explain this significant enrichment. Necsulea *et al*. [29] hypothesised that the differential methylation status of the silenced and active alleles of the monoallelically expressed genes may somehow be responsible for the increased recombinant hotspots in these genes. This begs the question whether the same mechanisms may be responsible for these recombinant hotspots as well as the CNV breakpoints?

The gene functional annotation clustering highlighted that many type III genes produce proteins that contain extracellular domains which agrees with Gimelbrant *et al.* [14]who found many monoallelically expressed genes were involved in cell to cell signalling. These domains are known to have evolved through the evolutionary process of exon shuffling [30] where whole exons and domains are duplicated or deleted within a gene. It is interesting that this mechanism is currently in action in the human genome.

Unlike type I genes, type III genes appear to be regulated by a number of potential dosage compensation mechanisms despite being present in just the wild type copy number. However, we agree with one of our reviewers who suggested that the complex nature of the different isoforms that may be produced might require more complex methods to regulate their expression.

The type II genes appear to fall between type I and III genes which makes them appear to be not significantly different to non CNV genes. This may be due in part to the miscategorisation of some type I or III genes as type II genes due to incorrect annotation of the UTRs or of the CNV breakpoint. However, the type II gene group is a real class as characterised by genes that become fusion genes for example in α-Thalassemia. Therefore, more research is required to fully understand this class of genes.

### Conclusion

Initially, this research set out to investigate how genes in copy number variable regions maintained expression levels similar to those of the wild type copy number. A new classification and characterization of CNV genes was required so that only the genes that fell entirely with in a CNV and that varied in copy number, would be used in studying gene expression. The characterisation was, originally based on the physical relationship of the gene with the CNV, however, it soon became apparent that this classification was biologically important. The genes in the three categories differ in their regulation and mutation rates as well as in the type of gene involved. Type I genes tend to be involved in immune response or sensory receptors while type III genes are involved in cell to cell signalling and type II genes are a complex mix of all three types.

While we still do not understand the homeostatic mechanisms involved in type I genes we do know that type III genes are more likely to be regulated by miRNAs and be monoallelically expressed. Type I genes appear to have evolved to avoid regulation by miRNAs as a necessity given their fast mutation rates and are also unlikely to be monoallelically expressed.

Further research is required as we still do not understand the mechanisms that underlie the homeostatic regulation of these CNV genes. Hopefully, new datasets such as histone and DNA modification scans will help shed light on why gene expression levels do not appear to be correlated with gene copy number. Disruption of these homeostatic mechanisms whether by CNVs or other mechanisms can often lead to disease. Therefore this is an important area for further research.

## Methods

All statistics were performed in R (http://www.R-project.org). Data manipulation was performed in Perl. Whilst, access to Ensembl version 53 was via the Perl API. [31] or Biomart [32]. The dN and dS values for the human/chimp one to one orthologues were obtained from Ensembl Compara as these have a high confidence of being true orthologues and compared with the Kolmogorov-Smirnov (KS) test. Note that the KS test is thought to be better for larger data sets such as those used here.

The ensembl gene ids for the three types of genes are available as supplemental information (Data S1).

### CNVs of HapMap Individuals

The co-ordinates for the CNVs were obtained from The Copy Number Variation (CNV) Project (http://www.sanger.ac.uk/humgen/cnv/data/), for the Redon *et al.* [33] and http://www.sanger.ac.uk/humgen/cnv/42mio/download_genotype_data.html for the Conrad *et al.* [1] data. The individuals used in this analysis were the unrelated individuals from Yoruba in Ibadan, Nigeria (YRI); Japanese in Tokyo, Japan (JPT); Han Chinese in Beijing, China (CHB); CEPH (Utah residents with ancestry from northern and western Europe) (CEU). It should be noted that there was no phenotypic information collected with sample sets therefore we cannot describe these cell lines as normal controls [34]. However, the presence of the same CNVs in different populations suggests that many of these CNVs are “normal” neutrally evolving genomic rearrangements.

### CNV Genes

The CNV co-ordinates are for the ncbi36 assembly of the human genome so the Ensembl API for ensembl version 53 (homo_sapiens_core_53_36o) [31] was used to map protein coding genes that overlapped with the CNV co-ordinates. Genes that were found to be entirely within the CNV breakpoints are classified as type I genes whilst those that overlap a CNV are type II. Some CNVs are complex with different individuals having different co-ordinates and copy numbers for CNVs in the same region of the genome. In these cases where the same gene may be classified as type I in one individual and type II in another, the gene was given the type I classification for the miRNA analysis but was counted as both in the expression analysis.

Note that only genes from the autosomal chromosomes are used.

### Expression Data

The expression data for the HapMap cell lines were obtained from ftp://ftp.sanger.ac.uk/pub/genevar [3]. Both the raw and the normalised (across all populations) data were downloaded. The expression results were filtered by probability (>0.95) as well as the variance of raw expression values. A value of >49 was used based on the work of Schuster-Böckler *et al.* [4], as it has been shown that probes with very low variance usually have low expression and are often at the limit of the sensitivity of the arrays [3], [4].

Ensembl genes without probes were not included in this analysis. Some probes represented more than one gene. in which case one probe was randomly taken to represent the gene. The expression ratio analysis was performed as described by Schuster-Böckler *et al.* [4]

### miRNA Targets

The experimentally verified miRNA target genes were downloaded from TarBase version 5 [19] and mapped to Ensembl gene identifiers.

The predicted miRNA target datasets came from miRGen version 3 [20]. We used the union and intersect datasets of miranda [20] http://www.microrna.org); PicTar miRNA target prediction from 4 way conservation with Human, Mouse, rat, and dog [22] http://pictar.mdc-berlin.de/) and TargetScanS (TargetScanS release 3.1. [18] http://www.targetscan.org/).

A simple χ^2^ analyses was used to test whether more or less genes than expected were predicted to be regulated by miRNAs. The expected values were calculated as expected value  =  Row total * Column total/Total for table.

The Kolmogorov-Smirnov test was used to compare the distributions for the numbers of predicted targets per gene for each gene type.

### Imprinted and Monoallelic Genes

The imprinted gene list was taken from the Catalogue of parent of Origin Effects database [13] and the monoallelically expressed genes were taken from the work by Gimelbrant *et al.* [13]. Again a χ^2^ test was to test whether these genes were enriched or not in the CNV genes.

### Cluster Analysis

The gene functional clustering was performed using DAVID, (The Database for Annotation, Visualization, and Integrated Discovery [35] from National Institute of Allergy and Infectious Diseases (NIAID), NIH, using lists of Ensembl identifiers.

## Supporting Information

Figure S1 Expression ratios for type II (A) and III (B) genes. Expression ratios (normalised expression) for each gene for duplicated and deleted genes as for figure 3.
(1.53 MB TIF)

Data S1 CNV genes. CNV genes used in this analysis.
(0.18 MB TXT)
