# Supplementary figures and images for: The Characterisation of Three Types of Genes that Overlie Copy Number Variable Regions
Source: PLoS One. 2011 May 26;6(5):e14814. doi: 10.1371/journal.pone.0014814 (PMC3102654; doi:10.1371/journal.pone.0014814)

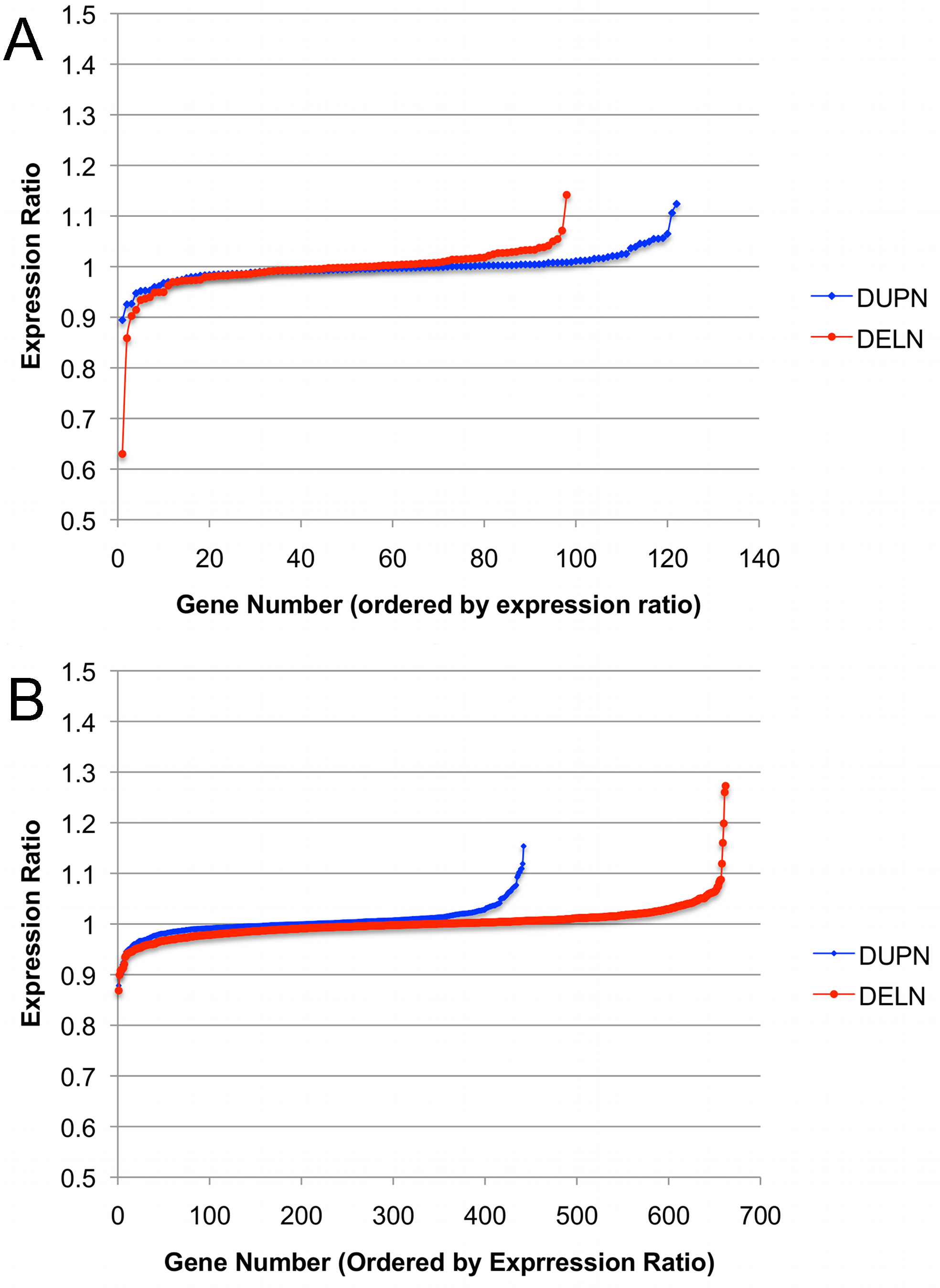

Supplement: Figure S1 — Expression ratios for type II (A) and III (B) genes. Expression ratios (normalised expression) for each gene for duplicated and deleted genes as for figure 3. (1.53 MB TIF) [file pone.0014814.s001.tif]
